# Modular 3D-printed fluorometer/photometer for determination of iron(ii), caffeine, and ciprofloxacin in pharmaceutical samples†

**DOI:** 10.1039/d3ra01281f

**Published:** 2023-04-17

**Authors:** Rafaela Silva Lamarca, João Pedro Silva, João Paulo Varoni dos Santos, Saidy Cristina Ayala-Durán, Paulo Clairmont Feitosa de Lima Gomes

**Affiliations:** a Department of Analytical Chemistry, Physical Chemistry and Inorganic Chemistry, National Institute for Alternative Technologies of Detection, Toxicological Evaluation and Removal of Micropollutants and Radioactives (INCT-DATREM), Institute of Chemistry, São Paulo State University (UNESP) Araraquara São Paulo 14800-060 Brazil paulo.clairmont@unesp.br

## Abstract

The demand for the development of portable and low-cost analytical devices has encouraged studies employing additive manufacturing techniques, such as 3D-printing. This method can be used to produce components such as printed electrodes, photometers, and fluorometers for low-cost systems that provide advantages including low sample volume, reduced chemical waste, and easy coupling with LED-based optics and other instrumental devices. In the present work, a modular 3D-printed fluorometer/photometer was designed and applied for the determination of caffeine (CAF), ciprofloxacin (CIP), and Fe(ii) in pharmaceutical samples. All the plastic parts were printed separately by a 3D printer, using Tritan as the plastic material (black color). The final size of the modular 3D-printed device was 12 × 8 cm. The radiation sources were light-emitting diodes (LEDs), while a light dependent resistor (LDR) was used as a photodetector. The analytical curves obtained for the device were: *y* = 3.00 × 10^−4^ [CAF] + 1.00 and *R*^2^ = 0.987 for caffeine; *y* = 6.90 × 10^−3^ [CIP] − 3.39 × 10^−2^ and *R*^2^ = 0.991 for ciprofloxacin; and *y* = 1.12 × 10^−1^ [Fe(ii)] + 1.26 × 10^−2^ and *R*^2^ = 0.998 for iron(ii). The results obtained using the developed device were compared with reference methods, with no statistically significant differences observed. The 3D-printed device was composed of moveable parts, providing flexibility for adaptation and application as a photometer or fluorometer, by only switching the photodetector position. The LED could also be easily switched, permitting application of the device for different purposes. The cost of the device, including the printing and electronic components, was lower than US$10. The use of 3D-printing enables the development of portable instruments for use in remote locations with a lack of research resources.

## Abstract 2

A low-cost modular 3D-printed fluorometer/photometer was built and applied to determine iron(ii), caffeine and ciprofloxacin in pharmaceutical samples.

## Graphical abstract

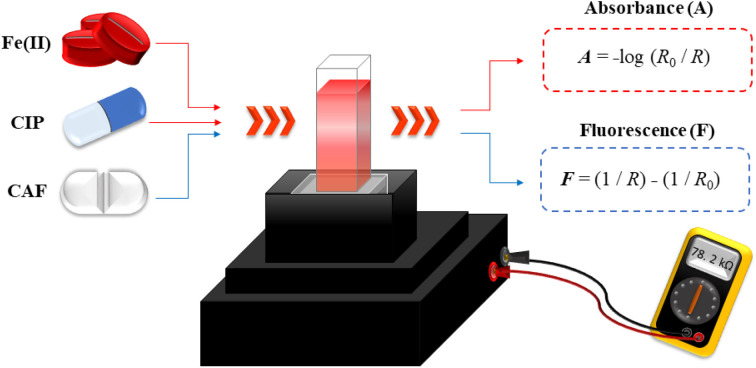

## 1. Introduction

In the last decades, there has been an increasing demand in analytical chemistry for the development of low-cost and portable devices that offer high analytical frequency. As a result, additive manufacturing and the development of 3D-printing has emerged as a highly promising technology for use in different research areas.^1^

3D-printing can be used to manufacture objects with specific characteristics, according to the required need, enabling prototypes to be produced easily, in a few steps and at low-cost. Furthermore, the layout can be modified and adjusted, without any requirement for the use of complex manufacturing processes and expensive equipment.^2,3^

The first step of the 3D-printing process consists of drawing the intended object, commonly employing computer-aided design (CAD) software. After this step, the CAD drawing is converted into a Standard Triangle Language (STL) file compatible with the printer used. The STL file is converted into sequential 2D layers, called a G-code file. The 3D-printer then produces the object, based on the information present in the G-code.^4^

Many research areas already use the benefits of 3D-printing. In analytical chemistry, applications have included the development of sensors/biosensors, electrodes, microfluidic devices, SPE columns, spot tests, and low-cost devices for educational purposes.^2,3,5–10^ In medicine, the use of 3D-printing is growing in different areas of research, such as the development of solid oral pharmaceutical formulas and the controlled release of drugs for the treatment of diabetes.^11,12^ In industry, in the so-called fourth industrial revolution, 3D-printing is widely used in the production of robotic components, as well as in the manufacture of various stainless steel parts.^13^

In analytical chemistry, the 3D-printing techniques used include selective laser sintering (SLS), selective laser melting (SLM), laser-assisted stereolithography (SLA), digital light processing (DLP), and fused deposition modeling (FDM).^4^

FDM 3D-printing is based on the extrusion of heated thermoplastics, such as polylactic acid (PLA), acrylonitrile-butadiene-styrene (ABS), and Taulman Tritan high-tensile polyester (Tritan). This technique involves the layer-by-layer deposition of the heated thermoplastic, until the intended object is formed.^2,4^ The temperature commonly used to manufacture items varies in the range from 230 to 285 °C, depending on the thermoplastic material employed.^4,14^ It is known that ABS filaments are more ductile, compared to PLA and Tritan. However, PLA and Tritan are more rigid, stronger, non-brittle, and provide greater mechanical resistance to traction. Tritan is more expensive than PLA, but has the advantage that it has high chemical resistance to acids, bases, and water, making it suitable for many applications.^14^ The preference for the use of PLA or Tritan in the printing of 3D parts lies in the fact that these materials are (bio)polyesters that are biodegradable, environmentally friendly, and safe to use. In addition, they can be produced on a large scale, at relatively low-cost.^4,14^

When spectrometric/fluorometric systems are used, an important parameter to consider is the type of radiation source. Currently, LED technology shows promise in many different fields, including the use of LEDs as radiation sources, due to the advantages of high brightness, low energy consumption, high conversion of electrical power to light, and almost zero heat generation.^15^ The use of LED-based devices is growing in chemistry, where they are used in a variety of applications, such as the detection of gas molecules, sensors based on absorbance, and the replacement of conventional light sources.^16^

The main objective of this work was to develop a simple, low-cost, modular light-emitting diode (LED) photometer/fluorometer, based on FDM 3D-printing. The modular 3D-printed device could be operated with the photodetector in different positions, obtaining two different configurations, as a photometer or a fluorometer. The radiation source could also be easily switched, permitting adaptation of the system according to the needs of the analyst. The 3D-printed device was applied for the determination of iron ions, caffeine, and ciprofloxacin in pharmaceutical formulations. Caffeine (CAF) is the most consumed psychoactive in the world,^17^ found in medicines, teas, coffees, and energy drinks. The use of CAF is associated with improvements in mood and physical performance, in addition to stimulating brain function.^18^ The monitoring of this compound in beverages is necessary, since it can have mildly addictive effects in users.^19^ Ciprofloxacin (CIP) is an antibiotic routinely used in the treatment of a wide variety of bacterial diseases.^20^ Fe(ii) is used in cases of anemia caused by deficiency of iron, especially in women of reproductive age and children. The applicability of the 3D-printed device was compared to the traditional techniques of HPLC and spectrophotometry.

## 2. Experimental

### 2.1 Photometer/fluorometer conception and construction

**Fig. 1:**
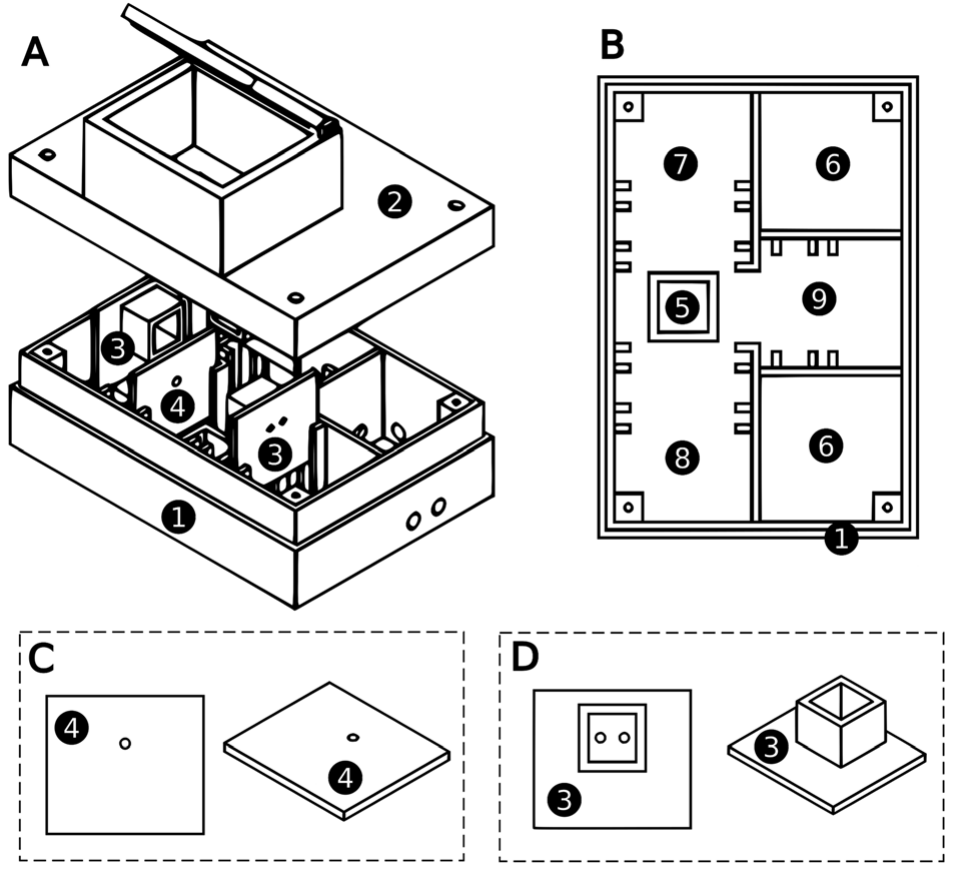
Schematic representation of the photometer/fluorometer constructed by the 3D-printing technique: perspective view (A); top view of the lower compartment (B); detail of the supporting parts for the light source and the detector (C); shield used to attenuate and focus part of the radiation (D). Legend: (1) lower compartment; (2) upper part (cover); (3) support parts for the light source and detector; (4) shield used to attenuate and focus part of the radiation; (5) compartment to hold the cuvette; (6) compartments used to contain wires and electronic components; (7) compartment to hold the light source; (8) compartment to hold the detector used in photometric experiments; (9) compartment to hold the detector used in fluorometric experiments.

The photometer/fluorometer device was drawn using FreeCAD v. 0.18, an open-source 3D CAD software package. The device consisted of two main parts. The base compartment had a space for a cuvette and fittings for smaller parts, such as light source and detector compartments, wires, and electronic connectors. The top lid enabled the device to be closed, avoiding the interference of external light during the analysis. A schematic drawing of the device is presented in Fig. 1, where the legend provides a brief description of all the device components. As can be seen, the device consisted of modules, enabling its assembly in different configurations. It was possible to switch the LEDs used as light sources, as well as the shields containing slits with varied diameters. Furthermore, the device could operate as a photometer or fluorometer, only requiring switching of the photodetector position (Fig. 1).

**Fig. 2:**
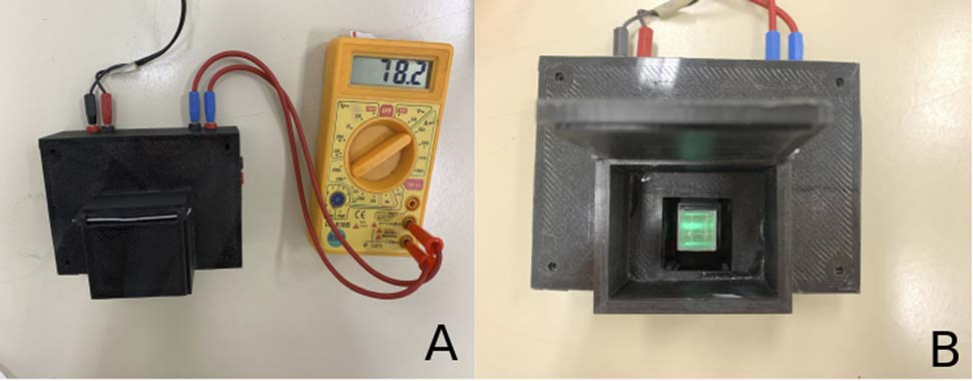
(A) Image of the 3D-printed fluorometer/photometer. (B) Picture of the 3D-printed device and the fluorescence reaction used to determine CAF in pharmaceutical samples.

All the plastic parts were printed separately by a 3D-printer (Core A3v2, GTMax3D), using Tritan copolyester as the filament material. The printing conditions were an extrusion temperature of 270 °C, bed temperature of 110 °C, and filament deposition print velocity of 70.0 mm s^−1^. Fig. 2A shows a photo of the fluorometer/photometer produced by 3D printing. Fig. 2B shows a picture of the 3D-printed device and the fluorescence reaction used to determine CAF in pharmaceutical samples.

Absorbance/fluorescence data were acquired using a DT830B digital multimeter. The photodetector was a light dependent resistor (LDR), where the incidence of light caused a change in the electrical resistance, which was measured using the multimeter. In the photometer mode, the light source and the detector were aligned at 180°, while in the fluorometer mode, the detector was positioned perpendicular (at 90°) to the light beam. The cuvette compartment was centered on the bottom of the device, in line with the radiation beam and detection compartments, as shown in Fig. 1B. Between the cuvette compartment and the radiation source support, there was a space to hold a shield with a small hole, which attenuated and focused the radiation before it reached the cuvette (Fig. 1A).

An electrical power supply with an output of 5.00 V and 2.00 A was connected to the radiation source. The modular design of the device allowed the use of different types of LEDs, with each having a specific operating condition, in terms of potential and electrical current. In general, ordinary LEDs, such as those employed here, require an electrical current of 20 mA for use under ideal conditions. For this purpose, a specific resistor was connected in series with each type of LED, enabling suitable conditions to be obtained.

**Fig. 3:**
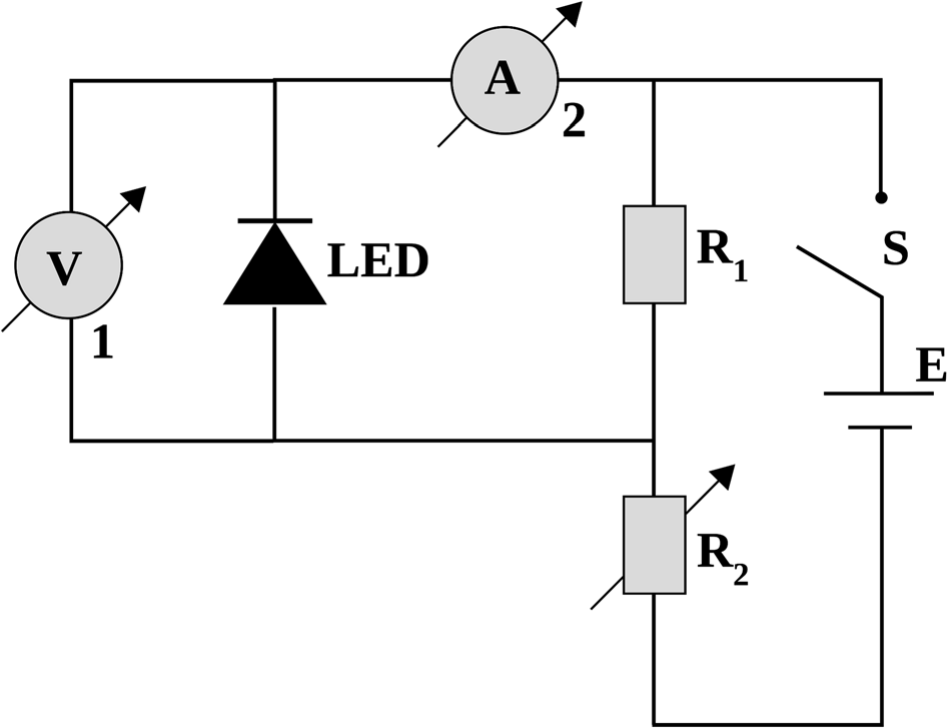
Simple electrical circuit used to obtain the LED current–potential curves: (S) on/off switch; (E) power source; (*R*_1_) fixed resistance (100 Ω); (*R*_2_) variable resistance; (1) voltmeter; (2) amperemeter.

The emission spectra of the LEDs used as light sources were obtained with a Red Tide USB 650 spectrometer (Ocean Optics). The current–potential curves for the LEDs were determined using the simple electrical circuit shown in Fig. 3, where the potentiometer (represented by an adjustable resistor) caused an ohmic drop in the circuit, keeping the LED potential between 1.50 and 4.00 V. The amperemeter measured the electrical current across the circuit, while the voltmeter provided the electrical potential at the LED.

### 2.2 Chemical reagents and solutions

Caffeine (CAF) (≥99%), ciprofloxacin (CIP) (≥98.0%), anhydrous ferric chloride, and 8-hydroxypyrene-1,3,6-trisulfonic acid trisodium salt (HPTS) (≥96.0%) were obtained from Sigma-Aldrich. Fe(NH_4_)_2_(SO_4_)_2_·6H_2_O (AFS) (99.0%) was obtained from PanReac. H_2_SO_4_ (98.0%), sodium acetate, hydroxylamine, and 1,10-phenanthroline were obtained from Synth.

Phosphate buffer solution (0.100 mol L^−1^, pH 6.30) was prepared in water, using dibasic potassium phosphate (K_2_HPO_4_) and monobasic potassium phosphate (KH_2_PO_4_), obtained from Sigma-Aldrich. A CAF stock solution (2000 mg L^−1^) was prepared in water, stored under refrigeration, and used for a maximum period of one week. A CIP stock solution (1000 mg L^−1^) was prepared in methanol, with addition of formic acid, and was stored under refrigeration. An HPTS stock solution (1.00 μmol L^−1^) was prepared in phosphate buffer and was used on the same day.

Aqueous solutions of H_2_SO_4_, CH_3_COONa, hydroxylamine, and 1,10-phenanthroline were prepared at concentrations of 10.0% (v/v), 10.0% (w/v), 10.0% (w/v), and 0.100% (w/v), respectively. The AFS standard was weighed out and H_2_SO_4_ was added in order to produce a stock standard solution with 100 mg L^−1^ of Fe(ii) and 1.00% (v/v) of H_2_SO_4_. Ultrapure water was used for the preparation of all the solutions.

### 2.3 Samples

Tablets of caffeine (210 mg), ciprofloxacin (500 mg), and ferrous sulfate (40.0 mg) were purchased at a pharmacy in Araraquara (São Paulo state, Brazil; 21°47′38′′S, 48°10′33′′W).

### 2.4 Photometric determination of Fe(ii)

Tablets of ferrous sulfate were used as samples for the determination of Fe(ii) using the 3D-printed photometer/fluorometer. According to the manufacturer's information leaflet, each tablet contained 40.0 mg of Fe(ii). For sample preparation, 6 tablets were weighed and ground in a mortar, after which a portion was weighed (∼0.1 g) and transferred to a 100 mL volumetric flask containing H_2_SO_4_ in sufficient quantity to yield a final solution with 1.00% (v/v) H_2_SO_4_, following completion of the volume with deionized water. For the photometry measurements, 1.00 mL of sample solution was transferred to a 50.0 mL volumetric flask, followed by addition of NaCH_3_COO, hydroxylamine, and 1,10-phenanthroline, as also used for the solutions employed for the analytical curve.^11^ A blue LED was used for this analysis, operating at 3.00 V and 15.0 mA. An analytical curve was constructed in the concentration range from 1.00 to 5.00 mg L^−1^.

As a comparative method, the samples were analyzed by a conventional spectrophotometric technique, employing a Shimadzu UV-1800 UV-vis spectrophotometer operated with UVProbe software. A calibration curve was constructed in the same concentration range described above. The sample solutions were the same as those evaluated using the 3D-printed device.

### 2.5 Photometric determination of CIP

The second analyte determined using the 3D-printed device was CIP. The analysis procedure was based on a previous study of pharmaceutical formulations, which employed a spot test and smartphone images.^10^ An aqueous solution of ferric chloride at 1.00% (w/w) was prepared and an aliquot of 350 μL of this solution was transferred to a 5.00 mL volumetric flask, followed by the addition of 40.0 μL of CIP solution. An analytical curve was constructed in the concentration range from 5.00 to 35.0 mg L^−1^, by adding, in 5.00 mL volumetric flasks, 350 μL of the aqueous solution of ferric chloride, an aliquot of the CIP stock solution, and water to complete the volume. The light source used was a blue LED (478 nm) operating at 3.0 V and 15 mA.

In this case, the analysis performed using the 3D-printed device was compared with an HPLC-UV-DAD method. The equipment used was a Shimadzu HPLC system equipped with an LC-20AT pump, a SIL-20A autosampler, and an SPD-M20A detector. A Kinetex EVO C18 reverse phase column (150 mm × 4.6 mm × 5 μm, 100 Å), kept at 30 °C, was used for the chromatographic separation, with isocratic elution using an 88 : 12 (v/v) mixture of an aqueous solution of 0.100% (v/v) formic acid and acetonitrile (also with 0.100% (v/v) of formic acid), at a flow rate of 1.00 mL min^−1^. The total analysis time was 5.00 min and the detector wavelength was set at 278 nm. The analytical curve was constructed using CIP concentrations in the range from 2.00 to 10.0 mg L^−1^.

### 2.6 Fluorometric determination of CAF

The experimental procedure for the analysis of CAF was based on a previously published method employing fluorescence to detect the compound in water samples, with polysulfonated pyrenes as reagent.^21^ An analytical curve was constructed in the concentration range from 50.0 to 300 mg L^−1^ by adding aliquots of 2000 mg L^−1^ CAF stock solution to 5.00 mL flasks, followed by fixed 25.0 μL volumes of HPTS solution to achieve a final concentration of 50.0 μmol L^−1^. The samples were obtained using five CAF tablets, which were weighed, ground, and solubilized in ultrapure water. The resulting solution was filtered through a crosslinked cellulose filter (0.45 μm), followed by transferring 500 μL of the filtrate and 25.0 μL of HPTS solution to a 5.00 mL volumetric flask. The flasks with the sample and standard solutions were completed with 0.100 mol L^−1^ phosphate buffer (pH 6.30). For the fluorescence quenching determination, a blue LED (478 nm) was used, operating at 3.00 V and 15.0 mA. The reaction between CAF and HPTS was allowed to occur for 10.0 min, before performing the fluorometric measurement with the 3D-printed device.

The reference method used to compare with the results obtained using the 3D-printed device was HPLC-UV-DAD, using the same equipment described above. Isocratic elution was performed with an eluent consisting of an 85 : 15 mixture of aqueous 0.100% (v/v) formic acid solution and acetonitrile (also containing 0.100% (v/v) formic acid), at a flow rate of 1.00 mL min^−1^. The chromatographic separation employed the same column described above, kept at 30 °C. The run time was 5.00 min and the detector wavelength was set at 270 nm. The analytical curve was constructed in the concentration range from 2.00 to 10.0 mg L^−1^. For the HPLC analysis, the procedures for sample preparation and the analytical curve were the same as described above, but without using HPTS (fluorophore).

### 2.7 Quality parameters of the methods for determination of CAF, CIP, and Fe(ii)

The limit of quantification (LOQ) was determined as the lowest concentration with a relative standard deviation (RSD%) lower than 15.0%, which was adopted as the first point of the analytical curve. The limit of detection (LOD) was the lowest detectable concentration that presented RSD% lower than 20.0%.^21,22^

The linearity of the developed methods was evaluated by ANOVA (95.0% confidence level). The analytical curves were performed in triplicate, in the concentration ranges 50.0–300 mg L^−1^ (CAF), 5.00–35.0 mg L^−1^ (CIP), and 1.00–5.00 mg L^−1^ (Fe(ii)).

Intra- and inter-day precision were also evaluated and expressed as RSD%. The intra-day precision was obtained by preparing analytical curves from analyses on the same day, in triplicate (*n* = 3). The inter-day precision was obtained by preparing analytical curves, in triplicate (*n* = 3), from analyses performed on different days and by different analysts.

Recovery assays were performed in triplicate (*n* = 3) spiking aliquots of the CAF, CIP stock solutions into the solubilized tablet samples. The labeled concentration for CAF, CIP considering the sample preparation were 210, 15.0 mg L^−1^, respectively. CAF and CIP were spiked with 40.0 and 10.0 into the samples solution. For Fe(ii) recovery was evaluated in five replicates (*n* = 5) using standard solution (data not shown) and tablet samples without spiking the stock solution into the samples. The tablet samples were diluted in order to obtain 2.00 mg L^−1^ of Fe(ii).

## 3. Results and discussion

### 3.1 Photometer/fluorometer concepts

**Fig. 4:**
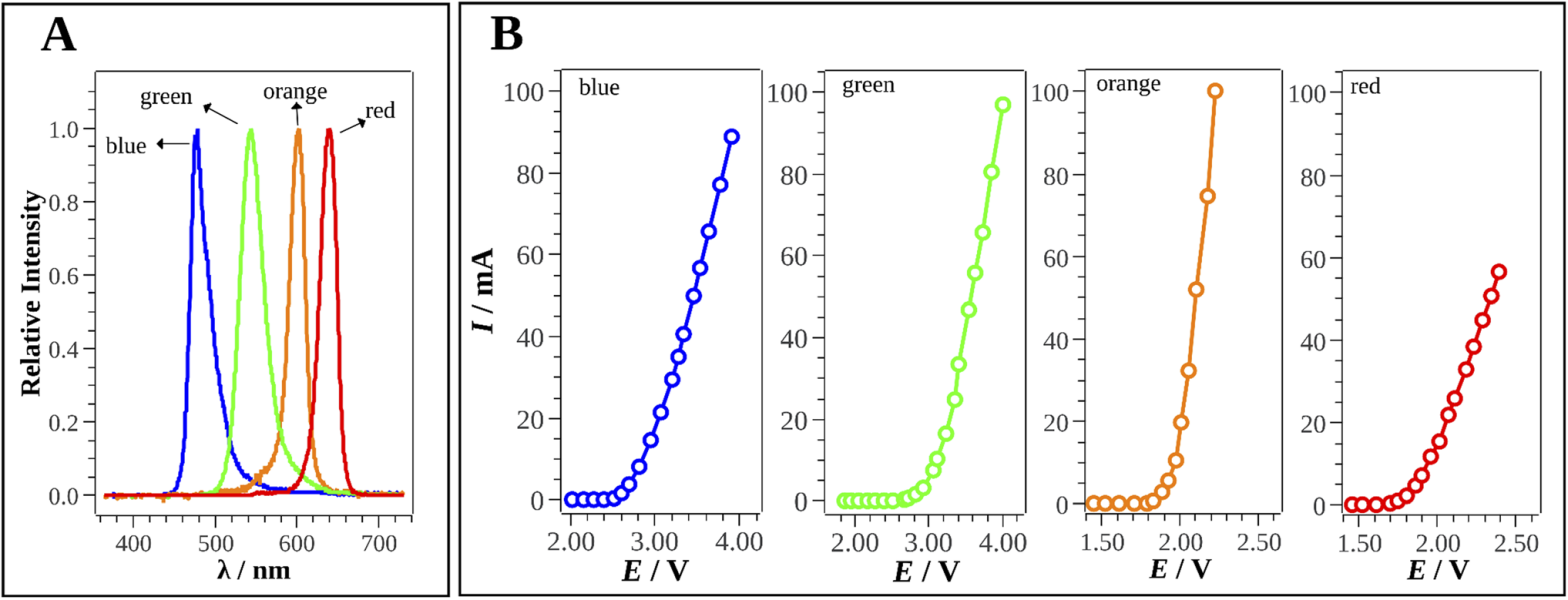
(A) LEDs emission spectra and (B) current–potential curves for blue, green, orange, and red LEDs.

The fact that the device was composed of switchable parts enabled easy substitution of the radiation source, according to the intended application. In addition, the photodetector could be easily switched and the 3D-printed device could be rapidly configured as a photometer or a fluorometer. According to the emission spectra of the LEDs (Fig. 4A), blue, green, orange, and red LEDs cover almost the entire visible light spectrum (450–680 nm). Fig. 4B shows the current–potential curves obtained for these LEDs.

Considering the 5.00 V delivered by the power supply to the photometer/fluorometer light source, an ideal current of 20.0 mA should be used. The potentials across blue, green, orange, and red LEDs are ∼3.00, ∼3.20, ∼2.10, and ∼2.00 V, respectively. In order to achieve these values would require resistors with electrical resistances of around 150, 160, 105, and 100 Ω, respectively. For practical reasons, 150 Ω resistors were used for the blue and green LEDs, while 100 Ω resistors were used for the orange and red LEDs, which enabled all the LEDs to perform satisfactorily.

The detector used in the 3D-printed device was an LDR. When exposed to light, this type of sensor exhibits a change in electrical resistance (*R*) that is inversely proportional to the intensity (*I*) of the light ^15^:

1 *I* = *k*/*R*

where, *k* is a proportionality constant. In photometry, according to the Lambert–Beer law, the absorbance (*A*) is related to the transmittance (*T*) by:

2 *A* = −log *T*

*T* is the ratio between the intensity of light that passes through a medium able to absorb light (*I*) and the intensity of light in the absence of absorption (*I*_0_). Combining eqn (1) and (2) gives:

3 *A* = −log *T* = −log(*I*/*I*_0_) = −log(*R*_0_/*R*)

where, *R*_0_ and *R* are the LDR electrical resistances in the absence and presence of light absorption, respectively. Therefore, for photometric measurement using the constructed device, the analyte concentration was proportional to −log(*R*_0_/*R*), where *R*_0_ was determined using a blank solution.

The analytical signal for the fluorometric measurement (*F*) is given by:

4 *F* = (1/*R*) − (1/*R*_0_)

where, 1/*R*_0_ is the signal for a blank solution.

The cost of the device was estimated based on prices valid on December 5th, 2022. The costs of the electronic components are listed in Table 1. It can be seen that less than US$10.0 was sufficient to build a modular 3D-printed fluorometer/photometer. This low cost of the device, achieved by the combination of simple electronic parts and 3D-printing, enables its use in laboratories with limited funding, as well as for teaching purposes in universities with a lack of research equipment.

**Table 1:** Estimated cost of the modular 3D-printed fluorometer/photometer used in this study

Electronic components	Price (US$)^a^
Green, red, orange, and blue LEDs	0.210
Resistances (2)	1.34
LDR	0.120
Digital multimeter	5.02

a Price estimated on https://amazon.com.

### 3.2 Photometric determination of Fe(ii)

The reaction between Fe(ii) and 1,10-phenanthroline, performed at pH 4.5–5.5, produced a red complex that was optically active for an extended time, with increase of the Fe(ii) concentration intensifying the observed color.^23^ The red Fe(ii)-phenanthroline complex has a maximum absorbance at 506 nm.^24^ The proposed reaction mechanism is shown in ESI† Fig. S1. According to Fig. 4A, the appropriate LED for determination of the Fe(ii)-phenanthroline complex was the blue LED (maximum emission at 478 nm).

### 3.3 Photometric determination of CIP

The charge transfer reaction between CIP and Fe(iii) ions leads to the formation of a yellow product (ESI† Fig. S2). As shown in Fig. S5,† increase of the CIP concentration was associated with an increase of the yellow color of the reaction products.^10^ This was measured experimentally using the multimeter, based on the increase of the electrical resistance values. The reaction product presents maximum absorption at 445 nm,^10^ so it could be determined using a blue LED (see Fig. 4A).

### 3.4 Fluorometric determination of CAF

The widely known reaction between caffeine and HPTS results in a solution with intense green color, involving the formation of a ground-state complex (ESI† Fig. S3). Increase of the CAF concentration leads to a decrease of the fluorescence intensity. In the 3D-printed device, this quenching of fluorescence was reflected by the increase of the electrical resistance measured using the multimeter.^21^ Since HPTS presents maximum absorption at 460 nm,^21,25^ the blue LED (maximum emission at 478 nm as shown in Fig. 4A) was used to promote the fluorescence phenomenon.

### 3.5 Quality parameters of the proposed methods

The linearity of the methods was evaluated by constructing analytical curves in the concentration ranges 50.0–300, 5.00–35.0, and 1.00–5.00 mg L^−1^, for CAF, CIP, and Fe(ii), respectively (ESI† Fig. S4–S6). The ANOVA results showed *F*_cal_ (4.50 × 10^−4^) < *F*_tab 95%_ (288) for CAF, *F*_cal_ (6.80 × 10^−6^) < *F*_tab 95%_ (374) for CIP, and *F*_cal_ (2.03 × 10^−4^) < *F*_tab 95%_ (168) for Fe(ii), indicating that there was no lack of fit for any of the methods developed using the 3D-printed device.^26^

The LOD values obtained were 25.0, 3.00, and 0.500 mg L^−1^, for CAF, CIP, and Fe(ii), respectively. The LOQ values were the first points of the analytical curves, corresponding to 50.0 mg L^−1^ for CAF, 5.00 mg L^−1^ for CIP, and 1.00 mg L^−1^ for Fe(ii). The intra-day precisions were 1.02% for CAF, 0.660% for CIP, and 0.330% for Fe(ii), while the inter-day precisions were 1.28% for CAF, 1.01% for CIP, and 1.64% for Fe(ii).

The obtained recovery (ESI† Table S1) were 99.2 and 100.5% for CAF, 98.4 and 100.7% for CIP, and 99.5% for Fe(ii). Table 2 summarizes the results obtained for all the methods using the 3D-printed device.

**Table 2:** Comparison of figures of merit for the 3D-printed fluorometer/photometer and reference methods

Figures of merit	Absorbance		Absorbance		Fluorescence	
	Fe(ii)		CIP		CAF	
	Device	Spectrophotometer	Device	HPLC-UV-DAD	Device	HPLC-UV-DAD
LOD/mg L^−1^	0.500	0.200	3.00	1.00	25.0	1.00
LOQ/mg L^−1^	1.00	0.500	5.00	2.00	50.0	2.00
Calibration curve	*y* = 1.12 × 10^−1^ [Fe(ii)] + 1.26 × 10^−2^*R*^2^ = 0.998	*y* = 1.96 × 10^−1^ [Fe(ii)] + 7.20 × 10^−3^*R*^2^ = 0.997	*y* = 6.90 × 10^−3^ [CIP] − 3.39 × 10^−2^*R*^2^ = 0.991	*y* = 3.09 × 10^5^ [CIP] + 6.14 × 10^4^*R*^2^ = 0.990	*y* = 3.00 × 10^−4^ [CAF] + 1.00 *R*^2^ = 0.987	*y* = 4.54 × 10^4^ [CAF] + 1.36 × 10^4^*R*^2^ = 0.998
Inter-day precision/RSD%	1.64	—	1.01	—	1.28	—
Intra-day precision/RSD%	0.330	—	0.660	—	1.02	—

### 3.6 Application of the proposed methods using pharmaceutical samples

After confirming that all the quality parameters were satisfactory, the 3D-printed device was used to determine the concentrations of CAF, CIP, and Fe(ii) in samples of pharmaceuticals. For CAF, the concentration stated on the packaging was 210 mg per tablet, while the result obtained with the developed methodology was 211 mg per tablet, in good agreement with the declared value. The concentration determined by the reference method was 215 mg per tablet. A *t*-test (95% confidence level) was applied, assuming equivalent variances, and no statistically significant difference was found between the new method and the reference method (*p*-value (0.40) > *α* (0.05)).^26^

For CIP, the concentration stated on the packaging was 500.0 mg per tablet, while the average value determined using the 3D-printed device was 503.4 mg per tablet, in agreement with the declared value. The concentration determined by the reference method was 510.0 mg per tablet. Application of the *t*-test (95% confidence level) showed no significant difference between the developed method and the reference method (*p*-value (2.77) > *α* (0.05)).

For Fe(ii), the concentration declared on the packaging was 40.0 mg per tablet. The average values obtained using the 3D-printed device and the spectrophotometric method were 39.1 and 39.3 mg per tablet, respectively, with no statistically significant difference between the methods, according to the *t*-test at the 95% confidence level (*p*-value (0.956) > *α* (0.05)).

Different studies in analytical chemistry using 3D-printed devices are listed in Table 3. Sulfate and quinine were determined in tonic water drink samples using a portable fluorometer constructed using PLA filament. Red and blue LEDs were used as light sources and the analysis results were obtained in real time or within 5 min.^27^

**Table 3:** Studies in analytical chemistry using 3D-printed devices

Device type	Filament material	Light source	Analytes	LOD/mg L^−1^	LOQ/mgL^−1^	Sample matrices	Sensing	Analysis time/min	Cost/US$	Ref.
Fluorometric/photometric	PLA	Blue LED	Quinine, sulfate	0.13, 0.11	0.43, 0.38	Tonic water drink samples	Smartphone camera	Instantly	a	27
Fluorometric	PLA	Blue LED	Caffeine	30	100	Commercial beverage and pharmaceutical samples	Smartphone camera	4	7.50	25
Photometric	a	Adjustable LED	Fe(ii), hypochlorite	2.95, 0.15	9.84, 0.50	No matrix	Webcam	a	a	28
Fluorometric	Polylactide	Blue, red, green LED	Quinine, riboflavin	0.14, 0.008	0.48, <0.1	Beer, white wine, energy drinks, and multivitamin supplement	Smartphone camera	Instantly	a	29
Fluorometric/photometric	Tritan	Blue LED	Caffeine, ciprofloxacin, Fe(ii)	25.0, 3.00, 0.500	50.0, 5.00, 1.00	Pharmaceutical samples	LDR	Instantly	7.00	This work

a Not informed.

CAF was determined in commercial beverages and pharmaceuticals using a smartphone camera coupled to a PLA 3D-printed box with a white LED as the radiation source, coupled to a smartphone camera to acquire the digital images. Also, as a radiation source a white LED was applied in order to determine CAF in commercial beverages and medicines. The analysis required only 4.00 min to be carried out at a cost of US$ 7.50.^25^

In other work, Fe(ii) and hypochlorite were determined through colorimetric reaction using a 3D-printed device. The 3D-printed device was developed to control the experimental conditions and reduce the errors caused by spurious radiation. An electronic circuit was used to control the LED light intensity and a webcam was used as the detector.^28^

Elsewhere, a 3D-printed fluorometer was built using a polylactide filament material, together with an RGB LEDs as the radiation source and a smartphone camera as the detector. This device was applied in fluorometric analysis of quinine, riboflavin, calcium, and phosphates in wine, beer, and multivitamin supplements samples. The fluorometric analysis was applied using this device with a smartphone camera as detector.^29^

In all the studies highlighted above, the analytical signal (fluorescence or absorbance) was obtained based on digital images acquired with a smartphone or a webcam. In contrast to these methods, the device developed here has the advantage of not suffering interference from spurious light during the analysis. Furthermore, digital images can vary according to the capture parameters, so control of the image acquisition protocol is essential to reduce oscillation of the analytical signal. Therefore, digital image methods are likely to suffer from lack of repeatability. These sources of errors are reduced using 3D-printed LED devices, increasing the method precision, as shown by the present results. In addition, the modular design of the device developed here enables it to be easily configured as a fluorometer or a photometer, only changing the LDR photodetector position. The LED can also be easily switched, according to the desired application. The same 3D-printed device can be used to measure fluorescence or absorbance, only moving parts present in the device.

This device could be used in remote locations by simply changing the power supply of the LEDs, using batteries or a power bank, since the digital multimeter already uses a battery. The device was shown to provide results very similar to those obtained using classical analysis methods such as HPLC or spectrophotometry. The proposed device produces minimal chemical waste and the analysis time is shorter than required for HPLC. The cost of the 3D-printed device is very low, when compared to an HPLC instrument, and its small power consumption allows it to be used in remote locations or in research laboratories with limited funding resources.

## 4. Conclusions

A new modular fluorometer/photometer device was developed using 3D-printing. The printed device had a total cost of US$7.00, including all the electronic parts (LED, multimeter, photodetector, and cables).

The device could be easily configured as a fluorometer or a photometer, by only switching the photodetector position. In both configurations, only 1.00 min was required to obtain the analytical signal. The device was constructed with detachable parts, providing flexibility since the radiation source could be easily switched, enabling the use of different types of LEDs, according to the desired analytical application.

Application of the *t*-test, at the 95% confidence level, showed no significant differences between the reference methods and the new methods using the 3D-printed fluorometer/photometer.

Therefore, this 3D-printed device has excellent potential for use in remote locations and in laboratories with limited research resources. The device could be easily adapted as a portable instrument, only requiring the replacement of the power supply by a battery or a power bank.

## Conflicts of interest

There are no conflicts to declare.

## Supplementary Material

RA-013-D3RA01281F-s001
