# Supplementary material for: Modular 3D-printed fluorometer/photometer for determination of iron(ii), caffeine, and ciprofloxacin in pharmaceutical samples
Source: RSC Adv. 2023 Apr 17;13(18):12050–8. doi: 10.1039/d3ra01281f (PMC10108832; doi:10.1039/d3ra01281f)
Supplement: RA-013-D3RA01281F-s001 [file RA-013-D3RA01281F-s001.pdf]

Electronic Supplementary Material (ESI) for RSC Advances.
This journal is © The Royal Society of Chemistry 2023

ELECTRONIC SUPPLEMENTARY MATERIAL

Modular 3D-printed fluorometer/photometer for determination of iron(II), caffeine, and ciprofloxacin in pharmaceutical samples

Rafaela Silva Lamarca[a], João Pedro da Silva[a], João Paulo Varoni dos Santos[a], Saidy Cristina Ayala-Durán[a], Paulo Clairmont Feitosa de Lima Gomes[a]

[a] Department of Analytical Chemistry, Physical Chemistry and Inorganic Chemistry, National Institute for Alternative Technologies of Detection, Toxicological Evaluation and Removal of Micropollutants and Radioactives (INCT-DATREM), Institute of Chemistry, São Paulo State University (UNESP), Araraquara, São Paulo, Brazil, 14800-060

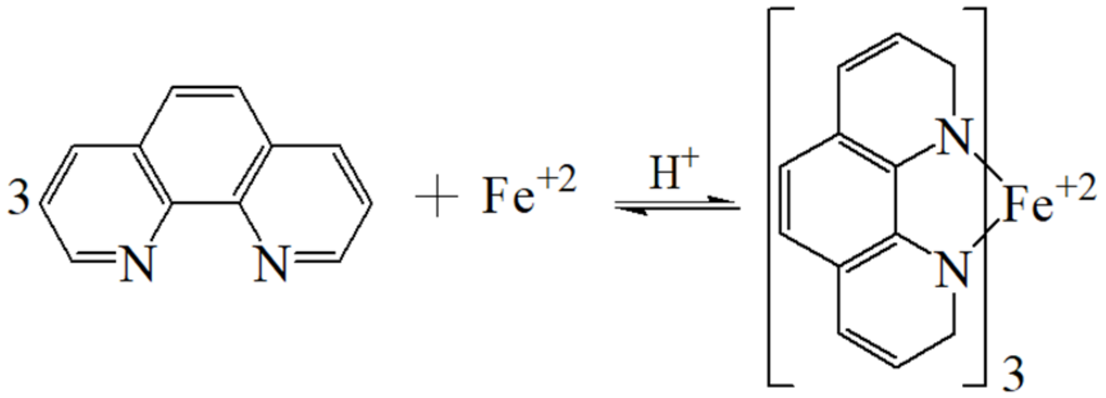

Figure S1 - The proposed reaction mechanism involving Fe(II) and 1,10-phenanthroline[1].

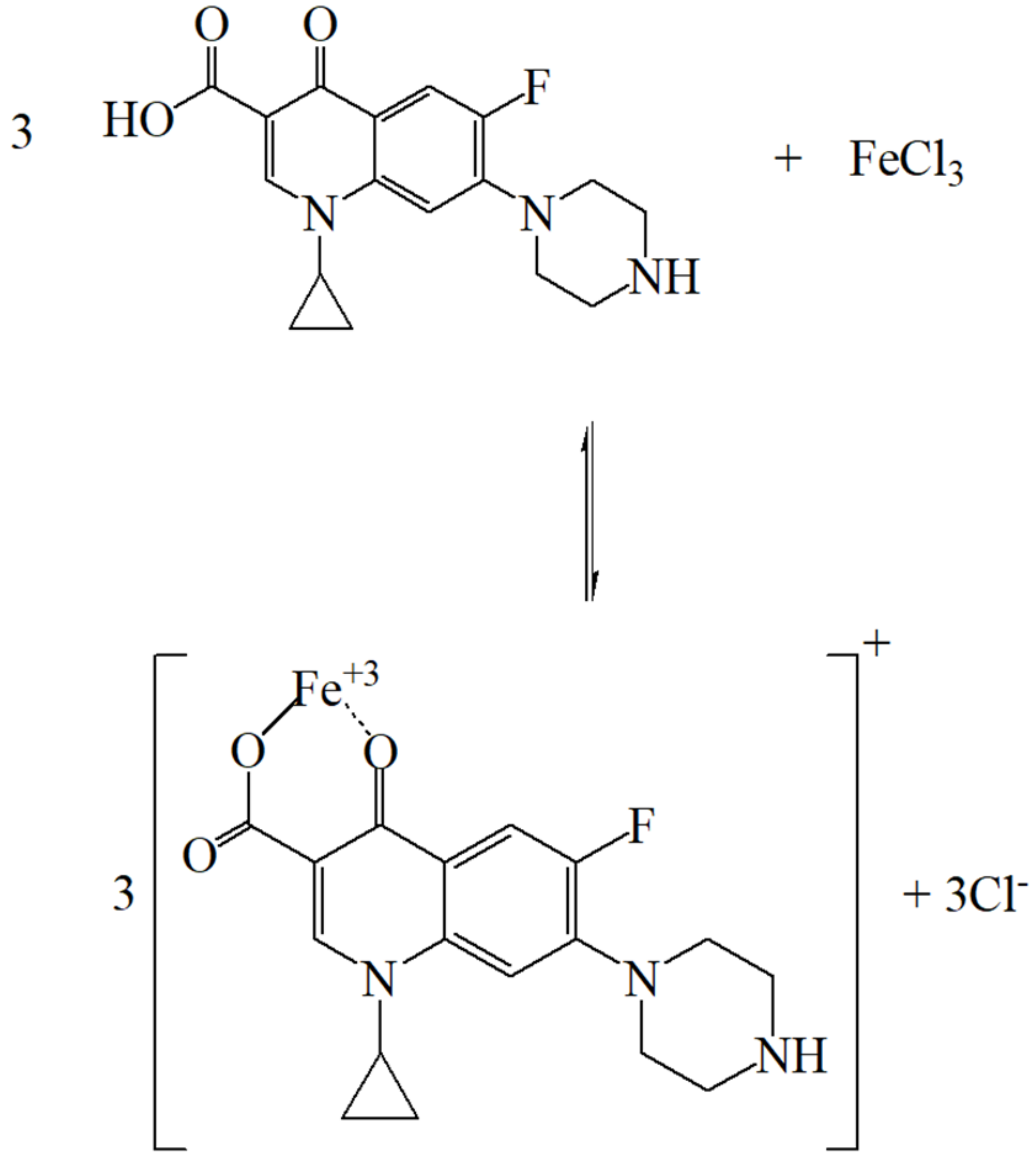

Figure S2 - The proposed reaction mechanism involving CIP and Fe(III) ions[2].

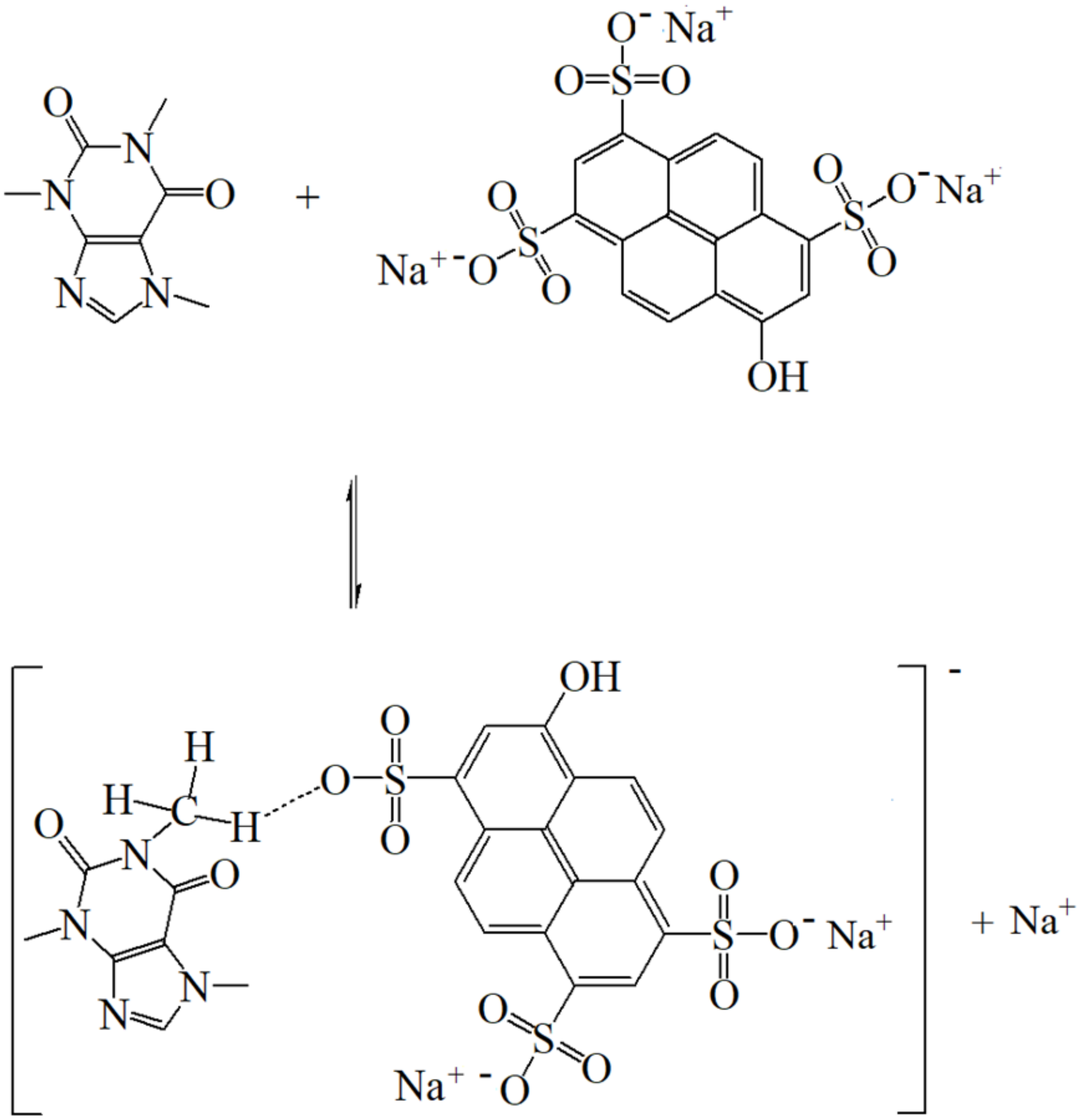

Figure S3 - The proposed reaction mechanism reaction involving caffeine and HPTS[3].

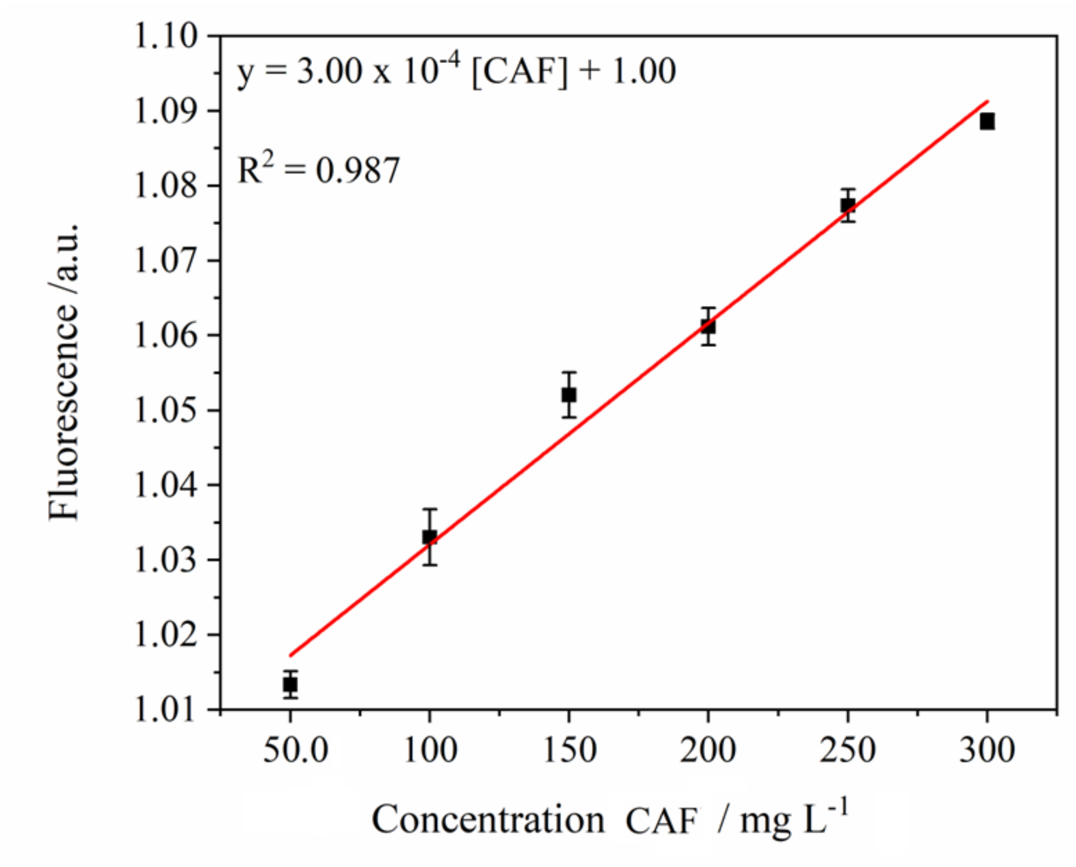

Figure S4 - CAF calibration curve obtained using the modular 3D-printed fluorometer.

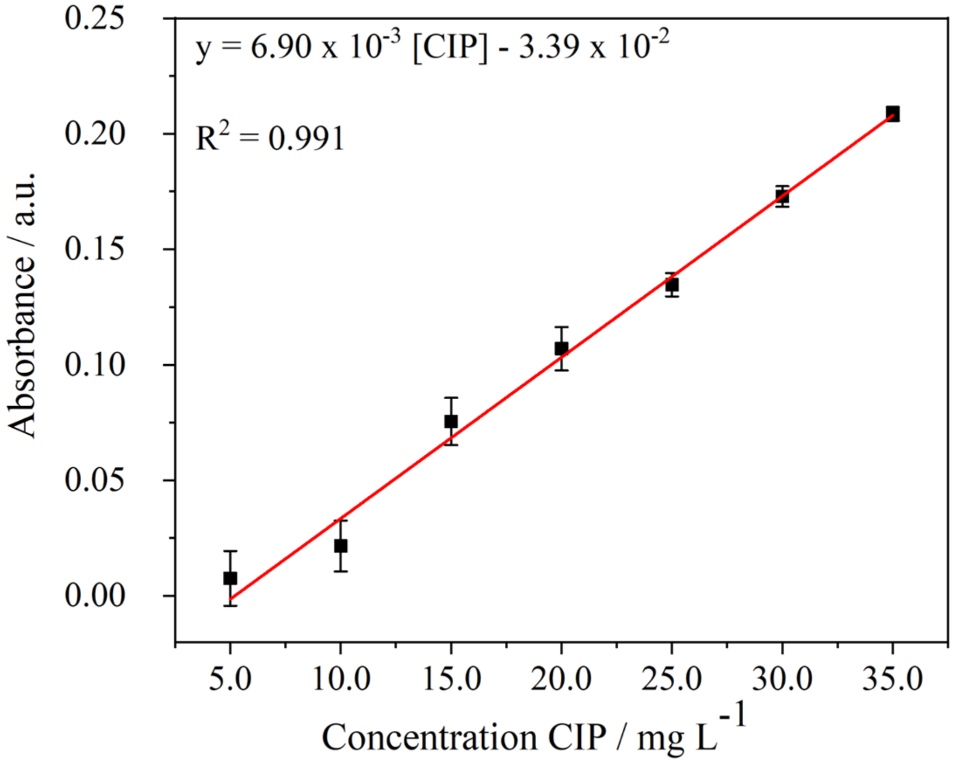

Figure S5 - CIP calibration curve obtained using the modular 3D-printed photometer.

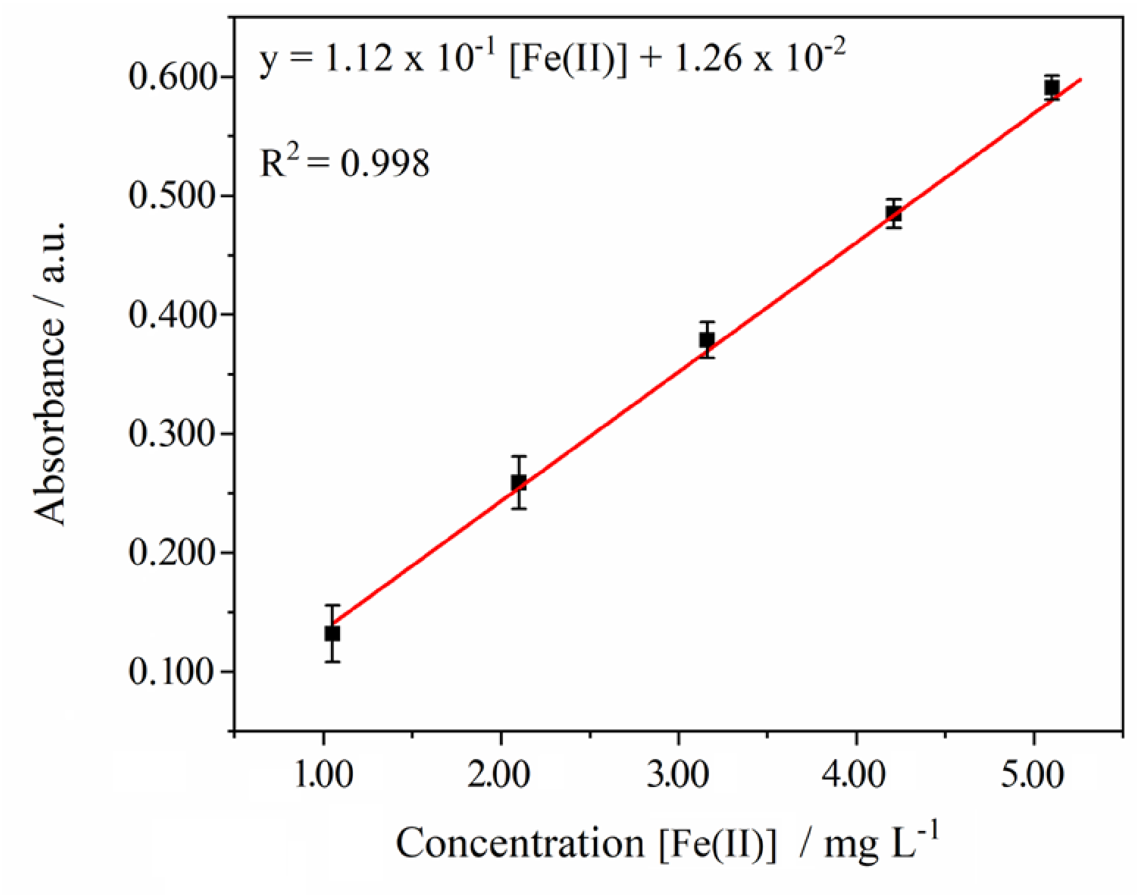

Figure S6 - Fe(II) calibration curve obtained using the modular 3D-printed photometer.

Table S1 - Recovery test performed in the CAF, CIP and Fe(II) tablets.

Analytes	Labeled concentration / mg L^{-1}	Found concentration / mg L^{-1}	Recovery	RSD / %	Spike / mg L^{-1}	Found after spike / mg L^{-1}	Recovery	RSD / %
**CAF**	210	211	100.5	5.50	40.0	248	99.2	3.30
**CIP**	15.0	15.1	100.7	1.80	10.0	24.6	98.4	1.30
**Fe(II)**	2.00	1.99	99.5	4.00	-	-	-	-

- **not evaluated.**

References

[1] Sandell, E. B. *Colorimetric Determination of Traces of Metals*, 3rd ed.; Interscience, Ed.; New York 1959.

[2] Sultan, S.M.; Suliman, F.E.O. Flow injection spectrophotometric determination of the antibiotic ciprofloxacin in drug formulations. Analyst 1992, 117 (9), 1523–1526, https://doi.org/10.1039/an9921701523.

[3] Rochat, S.; Steinmann, S. N.; Corminboeuf, C. and, Severin, K. Fluorescence Sensing of Caffeine in Water with Polysulfonated Pyrenes. *Chem. Commun. J.* **2011,** *47* (5), 10584–10586. https://doi.org/10.1039/c1cc13927d.
